# Reconfigurable Dual-Band SIW Bandpass Filter with Tunable Passbands and Enhanced Stopband Suppression

**DOI:** 10.3390/mi16111206

**Published:** 2025-10-23

**Authors:** Yongchae Jeong, Phanam Pech

**Affiliations:** 1 Division of Electronics and Information Engineering, Jeonbuk National University, Jeonju 54896, Republic of Korea; ycjeong@jbnu.ac.kr; 2 IT Convergence Research Center, Jeonbuk National University, Jeonju 54896, Republic of Korea; 3 Faculty of Electronics and Telecommunication, National Polytechnic Institute of Cambodia, Phnom Penh 12000, Cambodia

**Keywords:** bandpass filter, dual-band, substrate integrated waveguide, tunable passband

## Abstract

This paper presents a design approach for a dual-band substrate-integrated waveguide (SIW) bandpass filter (BPF) featuring passband tunability and wide-stopband characteristics. The proposed circuit is realized using half-mode (HM) SIW cavities loaded with tunable stopband resonators (TSRs). The TSRs are realized using transmission lines and varactor diodes. Passband tunability can be achieved by adjusting the supply voltage on the varactor diode. Wide-stopband characteristics can be achieved by integrating the defected microstrip structure into the proposed circuit. To validate the proposed concept, dual-band HM SIW BPFs with fixed and tunable passbands has been designed and fabricated. Based on the measurement results, the proposed circuits demonstrate high-frequency selectivity, with an attenuation level better than 20 dB and measured up to more than 40 GHz at the highest stopband. Moreover, the proposed tunable dual-band HM SIW BPF provides a passband tuning range of 240 MHz, measured from 4.88 GHz to 5.12 GHz for the first passband, and 310 MHz, measured from 6.19 GHz to 6.5 GHz for the second passband. Within the passband tuning range, the insertion loss varied from 1.7 dB to 2.2 dB for the first passband and 2.1 dB to 2.5 dB for the second passband.

## 1. Introduction

In modern wireless communication systems, the demand for high data rates and spectrum efficiency has led to the proliferations of multi-standard and multi-band operations. Multi-band bandpass filters (BPFs) have become essential components in such systems, enabling simultaneous or switchable operation across various frequency bands using a single circuit. The development of multi-band BPFs reduces circuit complexity, size, and cost by eliminating the need of separate filters for individual frequency bands. Furthermore, they facilitate better isolation, improved system integration, and enhanced performances in transceivers and front-end modules. Recently, substrate-integrated waveguides (SIWs) have provided a very attractive platform for designing low-cost and low-loss multi-band BPFs. The design of multi-band SIW BPFs on a single-substrate printed circuit board (PCB) had been presented in [1,2,3,4,5]. In [1], dual- and triple-band BPFs with inverter–coupled resonator sections had been discussed. In [2], a compact dual-band BPF using a half-mode (HM) SIW resonator and slot perturbation was designed. Similarly, compact planar dual-band SIW BPFs with widely separated passbands were presented in [3]. In [4], a dual-band SIW BPF was implemented by horizontally loading two identical pairs of complementary split-ring resonators (SCRRs) on the upper metal layer of the SIW. Similarly, the design of a dual-band BPF using a hybrid technique combined an SIW, spoof surface plasmon polariton (SSPP), and CSRR was introduced in [5]. Building on advancements in filter design, a new class of 3D multi-band BPFs based on SIW was developed in [6]. In addition, the design of compact multi-band BPFs based on multi-layer structure had been described in [7,8,9,10]. However, passband tunability and wide-stopband characteristics were not considered in these works, and the BPFs presented in [6,7,8,9,10] exhibited high circuit structure complexity, which necessitated advanced fabrication techniques and incurred high costs.

Recently, the design of ultra-compact single- and dual-band folded SIW filters with wide-stopband characteristics based on multiple embedded hybrid resonator modes was presented in [11]. The coupled-line based dual-band BPFs and filtering power dividers with reflectionless responses and enhanced upper-stopband suppression were reported in [12] and [13], respectively. In [14], a dual-mode composite right-/left-handed resonator with a fully symmetrical structure was introduced to design a high-order, high-temperature superconducting dual-band differential BPF. Although these design concepts were well presented, the passband tuning functionality was not considered. Moreover, the fabrication process might be difficult for the BPFs presented in [11]. In addition, the circuits presented in [12,13] were designed using coupled lines, which might be difficult to realize as the operating frequency increases to higher bands.

A simple approach to the design of a tunable dual-band BPF using a stub-capacitor-loaded HM SIW was reported in [15]. Furthermore, a dual-mode dual-band filter had been designed with dual-mode resonators with tuning functionality [16]. On the other hand, an amplitude tunable dual-band BPF with perfect absorption was described in [17], along with its sensing applications. However, the structure of the dual-band BPF in [15] may not be suitable for arbitrary filter-order (*n*) design, while the dual-band BPF presented in [16] exhibits unsatisfactory center-stopband performance. In [17], the circuit was realized using a five-layer structure from top to bottom: an upper metal pattern, a polyimide layer, a VO_2_ layer, a dielectric layer, and a lower metal pattern. As a result, the fabrication process was complicated and incurred high costs. Moreover, the stopband attenuation level and frequency selectivity of dual-band BPFs presented in these works are poor.

This paper proposes a new design approach for dual-band HM SIW BPFs with and without passband tunability featuring ultra-wide-stopband performances. The proposed dual-band BPFs are easily fabricated on a single-substrate PCB by utilizing HM SIW cavities and stopband resonators. Ultra-wide-stopband performances can be achieved through the integration of defected microstrip structures (DMSs) into the proposed circuit. The key novelties of this work lay in the integration of tunable stopband resonator (TSRs) and DMSs within the compact HM SIW configuration, enabling simultaneous passband tuning and ultra-wide-stopband suppression through a simple and efficient structure—an approach not previously reported in conventional dual-band SIW BPF designs.

## 2. Design Concepts and Equations

**Figure 1:**
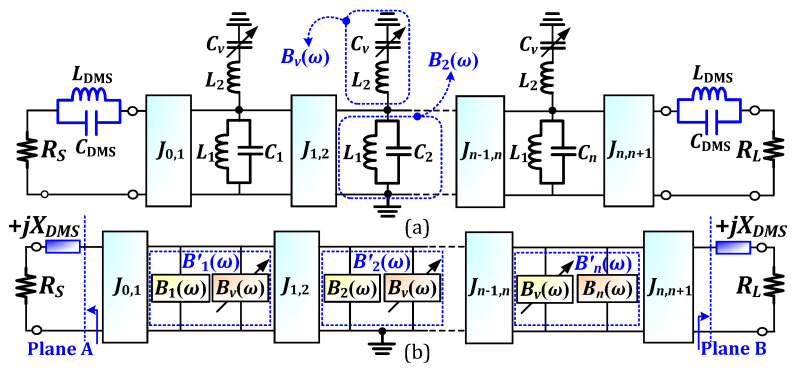
Proposed dual-band BPF: (**a**) initial equivalent circuit and (**b**) modification circuit based on (**a**).

Conventionally, a dual-band BPF can be designed by cascading a broadband BPF and a bandstop filter. But, in this work, the design of a dual-band BPF with the integration of bandpass and bandstop resonators is investigated. Figure 1a shows the equivalent circuit of the proposed tunable dual-band BPF with wide-stopband characteristics. It consists of *J*-inverters, shunt-parallel *L*_1_*C_i_* (*i* = 1, 2, ⋯, *n*), shunt-series *L*_2_*C_v_*, and *L_DMS_C_DMS_* resonators. The resonant frequency of the *L*_1_*C_i_* resonator is considered the center frequency (*f*_0_) of the broadband BPF. Similarly, the resonant frequency of *L*_2_*C_v_* resonator can be selected at any frequency within the bandwidth of the broadband BPF, and is denoted by *f_v_*. In contrast, the resonant frequency of *L_DMS_C_DMS_* resonator can be selected at any frequency within the highest stopband, particularly around 2*f*_0_. These resonators have distinct functionalities within the circuit. The *L*_1_*C_i_* resonator behaves as a bandpass resonator, while the *L*_2_*C_v_* resonator generates a stopband to split a broad passband into two passbands. Moreover, the *L_DMS_C_DMS_* resonator produces a transmission zero (TZ) at its resonant frequency to suppress the spurious responses within the highest stopband. As a result, a dual-band BPF with wide-stopband characteristics can be designed.

The susceptance parameters of *L*_1_*C_i_* and *L*_2_*C_v_* are denoted by *B_i_* and *B_v_*, respectively. As shown in Figure 1b, the equivalent susceptance of these resonators is denoted by *B*′*_i_*, which is equal to the parallel combination of *B_i_* and *B_v_*. On the other hand, *L_DMS_C_DMS_* ideally contains only a reactive component. Since it is cascaded with the port termination impedances (*Z_S,L_* = *R_S,L_* = 50 Ω) of the BPF, the impedances at planes A and B can be considered as *Z*′*_S,L_* = *R_S,L_* + *jX_DMS_* Ω. To match this reactive +*jX_DMS_*, the resonant frequencies of the first (*i* = 1) and last (*i* = *n*) resonators must be detuned, and can be calculated using (1):

(1) $$f_{S1,nL}=f_{0}\left[\sqrt{1+{\left(\frac{X_{DMS}\mathrm{FBW}}{2R_{S,L}g_{0,n}g_{1,n+1}}\right)}^{2}}+\frac{X_{DMS}\mathrm{FBW}}{2R_{S,L}g_{0,n}g_{1,n+1}}\right]$$

where *f_S_*_1_ and *f_Ln_* represent the detuned resonant frequencies of the first and last resonators, respectively. FBW is the desired fractional bandwidth of broadband BPF, while *g*_0_, *g*_1_, *g*_i_, and *g_n_* are the lowpass prototype values.

By choosing arbitrary values for *L*_1_ and *L*_2_, the capacitances *C*_1_ = 1/(2π*f_S_*_1_)^2^*L*_1_, *C_i_*_+1_ = 1/(2π*f*_0_)^2^*L*_1_, *C_n_* = 1/(2π*f_nL_*)^2^*L*_1_, and *C_v_* = 1/(2π*f_v_*)^2^*L*_2_ can be obtained. The slope parameters for the parallel *L*_1_*C_i_* resonators are defined as *b*_1_ = 2π*f_S_*_1_*C*_1_, *b_i_*_+1_ = 2π*f*_0_*C_i_*_+1_, and *b_n_* = 2π*f_nL_C_n_*. Similarly, the slope parameter for the series *L*_2_*C_v_* resonator connected in shunt can be determined as *b_v_* = 4π*f_v_C_v_*. Therefore, the equivalent slope parameter of these resonators can be determined as *b_eq,i_* = *b_i_b_v_*/(*b_i_*+*b_v_*). Then, the values of admittance *J*-inverters can be defined as (2):

(2) $$J_{01}=\sqrt{\frac{{\mathrm{FBW}b}_{eq,1}}{R_{S}g_{0}g_{1}}},J_{i,i+1}=\mathrm{FBW}\sqrt{\frac{b_{eq,i}b_{eq,i+1}}{g_{i}g_{i+1}}},\;\;J_{n,n+1}=\sqrt{\frac{\mathrm{FBW}b_{eq,n}}{R_{L}g_{n}g_{n+1}}}$$

The coupling coefficient (*K_i,i_*_+1_) of the resonators for the split passbands and the external quality factors (*Q_S_*_1,*nL*_) of the first and last coupled resonators are defined as follows [18]:

(3) $$K_{i,i+1}=\frac{J_{i,i+1}}{\sqrt{b_{eq,i}b_{eq,i+1}}},\;\;\;\;\;Q_{S1}=\frac{b_{eq,1}}{R_{S}J_{01}^{2}},\;\;\;\;Q_{nL}=\frac{b_{eq,n}}{R_{L}J_{n,n+1}^{2}}$$

**Figure 2:**
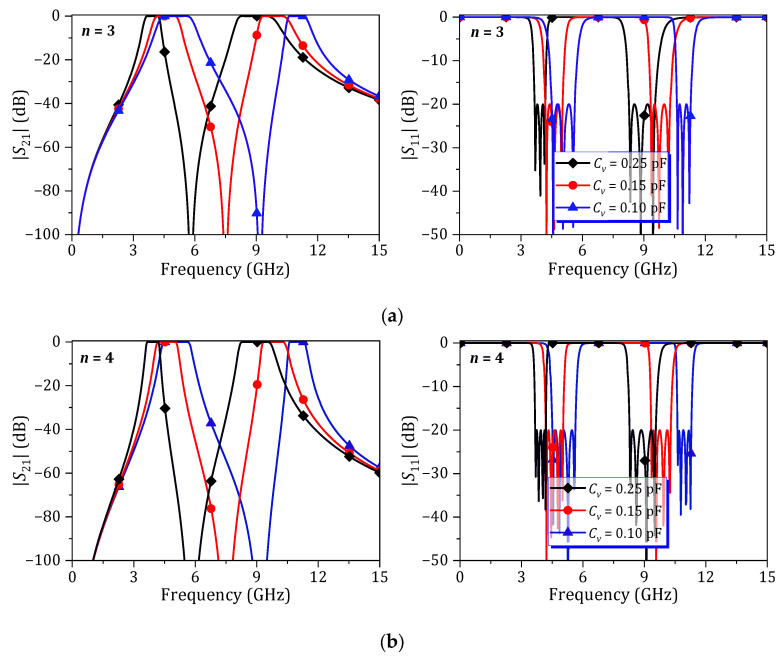
Simulated *S*-parameters of proposed dual-band BPF for different numbers of stages: (**a**) 3-stage and (**b**) 4-stage.

For this analysis, the dual-band BPFs are designed with an *f*_0_ of 6 GHz, |*S*_11_| of −20 dB, and an FBW of 30%. *L*_1_ = *L*_2_ = 0.2 nH is selected. The resonant frequency of the *L_DMS_C_DMS_* is set at 2*f*_0_, where *L_DMS_* = 0.8 nH and *C_DMS_* = 0.23 pF. Based on these values, *Z*′*_S,L_* = 50 + *j*40.7 Ω can be extracted at planes A and B. Using (1) and (2), the design parameters for the dual-band BPF can be calculated. Figure 2 shows the simulated *S*-parameters of the proposed dual-band BPFs for *n* = 3 and *n* = 4, with different *f_v_* values for the *L_2_C_v_* resonator. The results indicate that the proposed dual-band BPF can be designed with either odd- or even-order resonators. And frequency tunability is also achieved by varying the *f_v_* of the *L*_2_*C_v_* through the adjustment of *C_v_*. However, slight optimization is required for optimum return loss (RL) within both passbands.

For synchronously tuned coupling resonators, the *K_i,i_*_+1_ between two HM SIW resonators can be extracted from electromagnetic (EM) simulations using (4):

(4) $$K_{i,i+1}=\pm \frac{f_{p_{2}}^{2}-f_{p_{1}}^{2}}{f_{p_{2}}^{2}+f_{p_{1}}^{2}}$$

where *f_p_*_1_ and *f_p_*_2_ are the two split resonant frequencies.

Similarly, *Q_S_*_1_ and *Q_Ln_* can be extracted from the EM simulation and calculated using (5):

(5) $$Q_{S,L\_EM}=\frac{f_{S1,Ln}}{\Delta f_{\pm 3dB}}$$

where ∆*f*_±3dB_ is a 3 dB bandwidth.

## 3. Design and Implementation of Proposed Dual-Band SIW BPF

The proposed dual-band HM SIW BPFs were designed and implemented on Taconic-TLY substrate with *ε_r_* = 2.2, *h* = 0.508 mm and tan *δ* = 0.0009. As mentioned in [19], the design of RF circuits for future sixth-generation (6G) communication systems have recently attracted significant research attention. Although the specific operating bands for 6G have not been officially defined, various studies and industrial forecasts have identified promising candidate frequencies around 6–7 GHz [20] and 7–15 GHz [21,22,23]. Therefore, the circuit design parameters are given as *f*_0_ of 6 GHz, equivalent FBW of 12%, |*S*_11_| of −20 dB, and *n* of 3.

**Figure 3:**
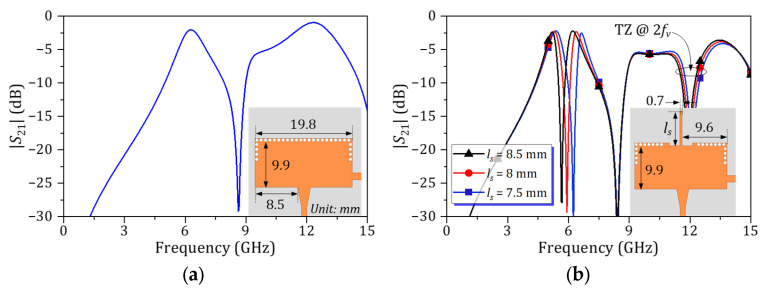
Simulated |*S*_21_| of (**a**) HM SIW resonator and (**b**) HM SIW loaded TL resonator.

The realization of several mode SIW resonators such as full-mode, half-mode, quarter-mode, and one-eight-mode with the equivalent circuit of LC-resonator are presented in [24,25,26]. Based on the approach presented in [25], the above-mentioned PCB information, and the desired *f*_0_, the physical dimension of the HM SIW resonator, such as width and length, can be defined. Figure 3a illustrates the simulated |*S*_21_| response of a stand-alone HM SIW resonator. A passband is observed at 6 GHz, while a spurious response appears, worsening around 12 GHz. Similarly, Figure 3b presents the |*S*_21_| characteristic of the HM SIW loaded with an open-stub transmission line (TL) resonator. This open-stub TL resonator, representing the *L*_2_*C_v_* resonator, has a resonant frequency that can be controlled by adjusting its length (*l_s_*). As shown, the stopband response produced by the open-stub TL resonator splits the single broad passband of the HM SIW resonator into two distinct passbands. Furthermore, this open-stub TL resonator also produces a TZ at approximately 2*f_v_*.

**Figure 4:**
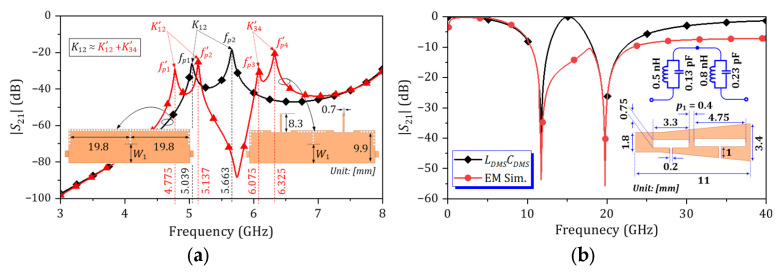
Characterization of (**a**) split resonant frequencies for HM SIW cavities, both with and without the inclusion of stopband resonators, at *W*_1_ = 6.65 mm. (**b**) Simulated |*S*_21_| characteristics and dimensions of asymmetrical DMSs.

Figure 4a shows the split resonant frequency characteristics of HM SIW cavities with and without open-stub TL resonators. By integrating the HM SIW cavity with an open-stub TL resonator, the two-split resonant frequencies become four. The coupling coefficient between two HM SIW resonators is denoted by *K*_12_. Similarly, the coupling coefficients resulting from the integration between HM SIW and open-stub TL resonators are denoted by *K*′_12_ and *K*′_34_. Using (4), it is evident that *K*_12_ is approximately equal to the sum of *K*′_12_ and *K*′_34_. This implies that a broad passband can be split into a dual-passband, with only a slight effect on the equivalent FBW. The FBW of the proposed circuit can be controlled by adjusting the width of the iris window (*W*_1_).

To enhance the stopband attenuation of the proposed dual-band HM SIW BPF, the asymmetrical DMSs are embedded in the circuit. Typically, increasing the number of DMS units with T-shape slot improves the attenuation level at the stopbands [27]. Therefore, this design utilizes double-DMSs with different resonant frequencies. Figure 4b illustrates the |*S*_21_| characteristics, equivalent model, and layout with dimensions for this double-DMS. The resonant frequencies of the individual DMS units are selected at 12 GHz and 20 GHz. The DMS units are separated by a spacing length (*p*_1_). As *p*_1_ decreases, the frequency response of the double-DMS more closely resembles that of its equivalent circuit. Based on this, *Z_S_*′ = *Z_L_*′ = 52.4 + *j*60.7 Ω are extracted at planes A and B. By applying (1), (2), and (3) sequentially, the reference values for *Q_eS,eL_* and *K_i,i_*_+1_ can be calculated.

**Figure 5:**
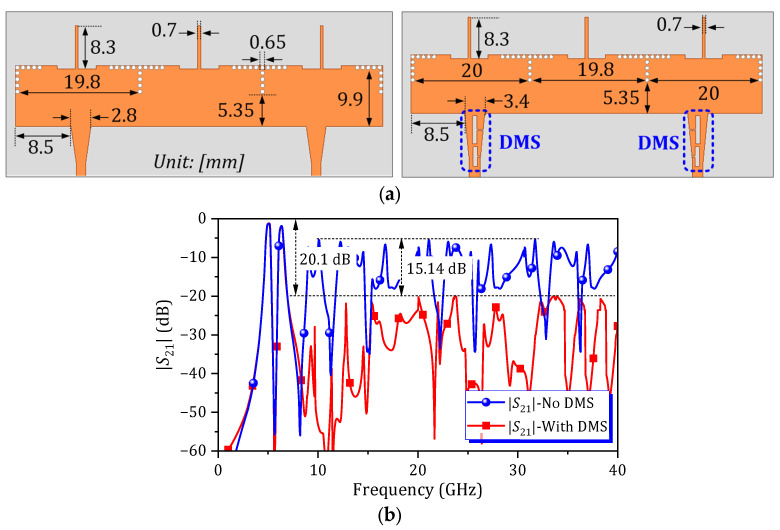
Dual-band HM SIW BPF with and without DMSs: (**a**) layout with dimensions and (**b**) simulated *S*-parameters.

Figure 5a displays the layout and dimensions of the dual-band HM SIW BPF, both with and without a DMS. A comparison of the *S*-parameters for these two configurations is presented in Figure 5b. By embedding the DMS at the input and output ends of the proposed circuit, the wide-stopband characteristic can be achieved with a minimum attenuation level of 20.1 dB. This represents a significant improvement of 15.14 dB compared to the dual-band SIW BPF without a DMS.

**Figure 6:**
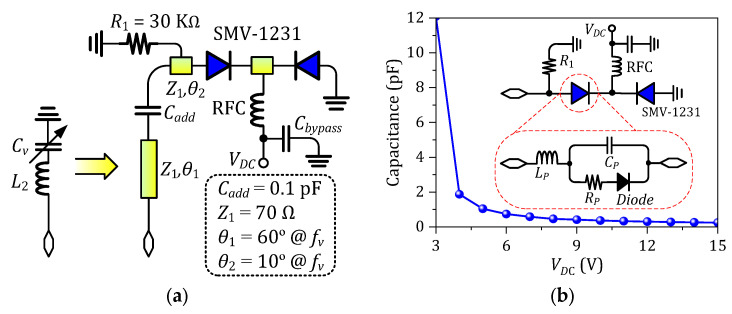
Stopband resonator for proposed tunable dual-band HM SIW BPF: (**a**) circuit configuration and (**b**) equivalent capacitances of two series SMV-1231 as a function of applied voltage (*V_DC_*).

To design a tunable dual-band HM SIW BPF, the *L*_2_*C_v_* resonator was realized using TLs and varactor diodes (SMV-1231) from Skyworks [28], as can be seen in Figure 6a. Figure 6b illustrates the capacitance variation in two series SMV-1231 as a function of the applied voltage (*V_DC_*), extracted at 6 GHz. The overall equivalent capacitance is decreased as *V_DC_* increases. For the proposed tunable dual-band HM SIW BPF, a small capacitance for *C_v_* is required. Therefore, the series connection of two SMV-1231 varactor diodes and *C_add_* is considered. *C_add_* is also operated as a DC-block capacitor. Slight optimization led to the selection of a TL with electrical parameters of *Z*_1_ = 70 Ω and *θ*_1_ = *λ*/6 = 60° at *f_v_* of 5.8 GHz. The other TLs with electrical parameters of *Z*_1_ and *θ*_2_ = 10° at *f_v_* are used as the soldering pad. When the TLs connected with *C_DC_* and varactor diodes, its resonant frequency must be within the desired equivalent bandwidth.

## 4. Simulation and Measurement Results

### 4.1. Dual-Band HM SIW BPF with Fixed Passband

**Figure 7:**
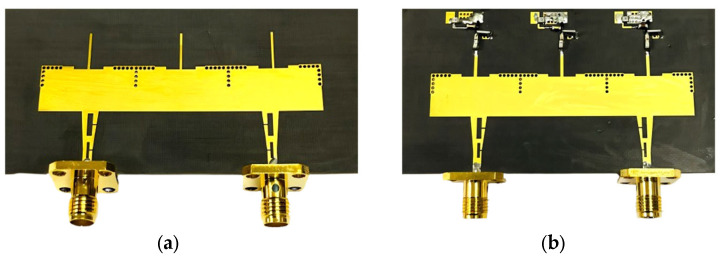
Photographs of fabricated circuits: (**a**) fixed dual-band SIW BPF and (**b**) tunable dual-band SIW BPF.

**Figure 8:**
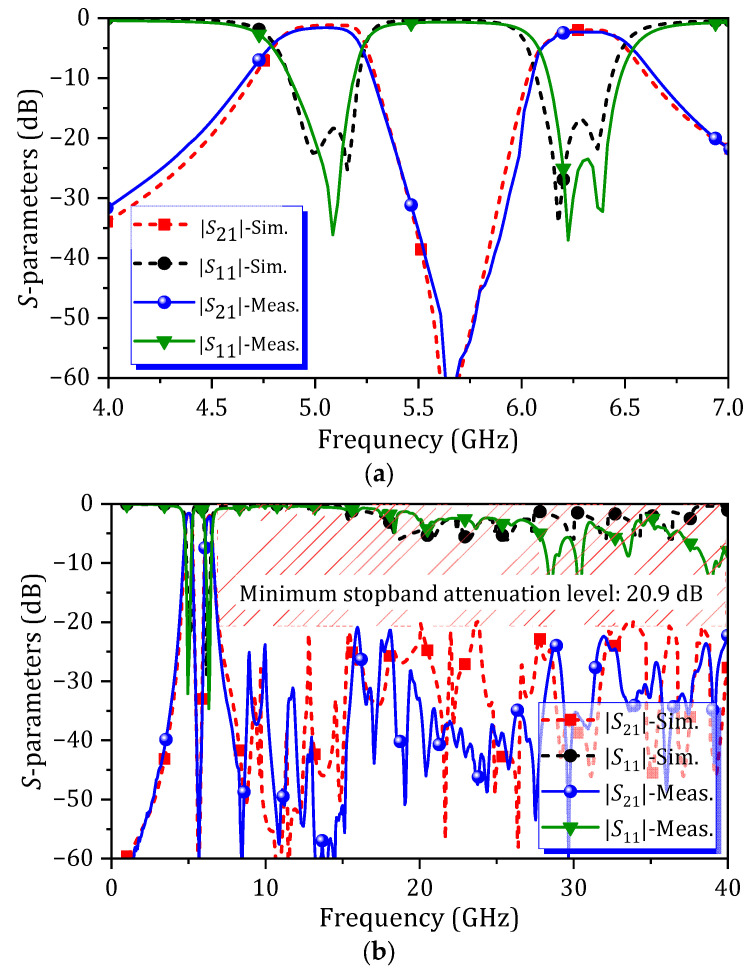
Simulated and measured *S*-parameters of fixed dual-band HM SIW BPF across different frequency ranges: (**a**) from 4 GHz to 7 GHz and (**b**) from DC to 40 GHz.

A photograph of the fabricated non-tunable dual-band HM SIW BPF is presented in Figure 7a. The comparison between simulated and measured S-parameter characteristics in narrow and wide frequency ranges are shown in Figure 8a and Figure 8b, respectively. For the first passband, the 3 dB bandwidth is 360 MHz, measured from 4.83 GHz to 5.19 GHz. For the second passband, the 3 dB bandwidth is 320 MHz, measured from 6.15 GHz to 6.47 GHz. The insertion loss (IL) of 1.6 dB is measured at 5 GHz for the first passband, and 2.3 dB is measured at 6.3 GHz for the second passband. A minimum attenuation level of 20.8 dB is measured across the range of 7.4 GHz–40 GHz. Furthermore, high isolation between both passbands is achieved, with a maximum attenuation level higher than 60 dB measured at 5.65 GHz.

### 4.2. Dual-Band HM SIW BPF with Passband Tunability

**Figure 9:**
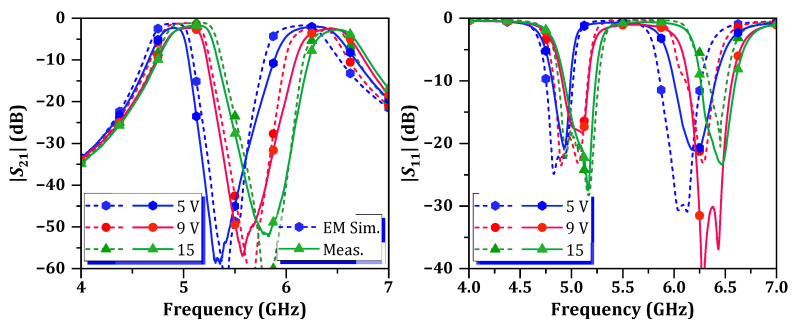
Simulated and measured *S*-parameters of proposed tunable dual-band HM SIW BPF.

**Figure 10:**
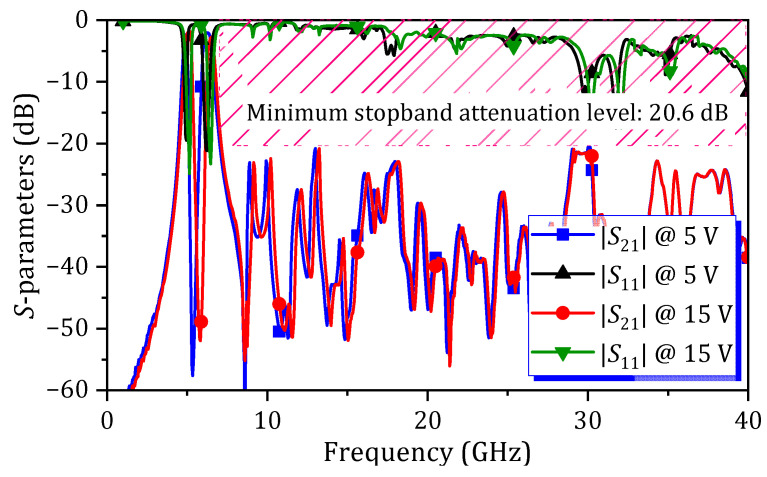
Measured *S*-parameters of the proposed tunable dual-band HM SIW BPF across the frequency range from DC to 40 GHz at *V_DC_* of 5 V and 15 V.

A photograph of the fabricated tunable dual-band HM SIW BPF is shown in Figure 7b. The comparison between simulation and measurement results of the *S*-parameters characteristics is presented in Figure 9. The *S*-parameters illustrate the characteristics of proposed tunable dual-band SIW BPF under *V_DC_* conditions of 5 V, 9 V, and 15 V. As observed, both the passband and center stopband are shifted as the *V_DC_* is varying. The passband tuning range for the first passband is 240 MHz (measured from 4.88 GHz to 5.12 GHz), and for the second passband it is 310 MHz (measured from 6.19 GHz to 6.5 GHz). The IL and RL measured at *f_c_* of each passband, according to the different *V_DC_*, are summarized in Table 1. Within the passband tuning range, the ILs of the first and second passbands vary from 1.7 dB to 2.2 dB and from 2.1 dB to 2.5 dB, respectively. Figure 10 presents the S-parameter characteristics of the proposed tunable dual-band HM SIW BPF, measured from DC to 40 GHz. A minimum attenuation level of 20.6 dB is observed from 7.08 to 40 GHz.

**Figure 11:**
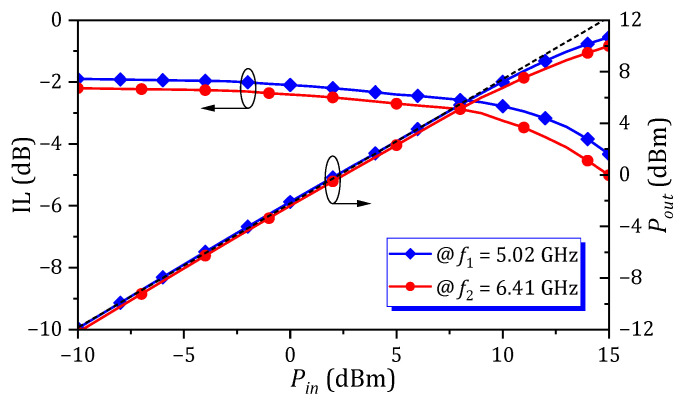
Measured power handling capability of proposed tunable dual-band HM SIW at the condition of *V_DC_* = 9 V.

The power-handling capability of proposed circuit is primarily limited by the nonlinearity of varactor diodes. Figure 11 shows the measured 1 dB compression point (*P*_1dB_) at 5.02 GHz for the first passband and 6.41 GHz for the second passband at the condition of *V_DC_* = 9 V. As can be seen, the IL is degraded as the input power (*P_in_*) increases. This degradation becomes significant when *P_in_* exceeds 10.5 dBm, while the *P*_1dB_ is approximately 11 dBm.

The electrical performances of the proposed tunable dual-band HM SIW BPF are compared with state-of-the-art dual-band SIW BPFs, as listed in Table 2. The dual-band SIW BPFs presented in [3,7,9] did not consider passband tunability, and their stopband performances and attenuation levels are poor. Furthermore, the dual-band SIW BPFs presented in [7,9] are realized with five copper layers, making their fabrication difficult and costly. Even though the tuning range (Δ*f*/*f*_0_) of the dual-band SIW BPFs presented in [11,12] are wider than our proposed circuit, the structure of dual-band SIW BPF presented in [11] may limit the ability to increase the number of resonator stages for improved frequency selectivity. Additionally, the frequency selectivity and stopband performance of the dual-band SIW BPF in [12] are significantly poorer than the proposed circuit.

## 5. Conclusions

This paper presents a new design approach for a dual-band HM SIW BPF featuring both passband tunability and wide-stopband characteristics. The dual-band response is achieved by integrating an HM SIW cavity with a stopband resonator. To ensure wide-stopband performance, DMSs were embedded at the input and output ends of the circuit. Based on measured electrical performances, the proposed concept successfully yields the dual-band SIW BPFs with high isolation between passbands, passband tunability, and wide-stopband characteristics. This circuit is well-suited for applications in multi-band communication systems. For future work, further enhancement of the passband tuning range can be explored to achieve greater frequency agility and adaptability for wider application scenarios.

## Figures and Tables

**Table 1:** Electrical performances of both passbands according to *V_DC_*.

	First Passband			Second Passband		
***V_DC_* (V)**	**5**	**9**	**15**	**5**	**9**	**15**
***f_c_* (GHz)**	4.88	5.02	5.12	6.19	6.41	6.5
**IL (dB)**	2.2	2.1	1.7	2.1	2.4	2.5
**RL (dB)**	19.2	18	20.9	20.9	31.3	22.6

**Table 2:** Electrical performance comparison with state-of-the-art dual-band SIW BPFs.

Ref.	*f*_1_/*f*_2_ (GHz)	3 dB-FBW (%)	IL (dB)	Δ*f*/*f*_0_ (%)	Copper Layers	Possibility for *n*-Stage	Rejection	Size (*λ_g_* × *λ_g_*)
[3]	5/7.5	5.46/4.75	1.7/2.3	No	2	Yes	≈20 dB up to 1.7*f*_1_	1.65 × 0.93
	5/8.5	6.26/7.75	2/1.8	No	2	Yes	≈20 dB up to 2*f*_1_	1.31 × 0.84
[7]	8.71/10.1	1.9/2.7	2.8/2	No	5	Difficult	≈20 dB up to 1.3*f*_1_	0.87 × 0.87
[9]	6.97/7.46	2.04/3.26	2.8/2.3	No	5	Difficult	≈20 dB up to 1.3*f*_1_	0.83 × 0.83
[11]	1.85~2.67/ 3.84~5.34	14.9~19.2/ 10.3~17.2	1.3~2.3/ 1.7~3.3	36.4 32.7	2	Difficult	≈20 dB up to 3*f*_1_ */ ≈10 dB up to 1.8*f*_1_ **	0.19 × 0.15
[12]	3.26~3.47/ 5.47~6.13	N/A	0.2~2.9/ 0.1~2.1	6.2 11.4	2	Yes	≈10 dB up to 2*f*_1_ */ ≈12 dB up to 1.2*f*_1_ **	N/A
**This** **Work**	**4.88~5.12/** **6.19~6.5**	**3.2~6.64/** **2.62~5.8**	**1.7~2.2/** **2.1~2.5**	**4.8** **4.9**	**2**	**Yes**	≈20.6 dB up to 8.2*f*_1_	1.13 × 0.53

Note: *: at the condition of tuning the first passband; **: at the condition of tuning the second passband; *λ_g_*: guided wavelength at *f*_0._

## Data Availability

The original contributions presented in this study are included in the article. Further inquiries can be directed to the corresponding author.

## Abbreviations

The following abbreviations are used in this manuscript:

BPF	Bandpass filter
CSRR	Complementary split-ring resonator
DMS	Defected microstrip structure
HM	Half-mode
IL	Insertion loss
PCB	Printed circuit board
RL	Return loss
SIW	Substrate integrated waveguide
TL	Transmission line
TSR	Tunable stopband resonator
TZ	Transmission zero
